# Evidence that C/EBP-β LAP Increases Fat Metabolism and Protects Against Diet-Induced Obesity in Response to mTOR Inhibition

**DOI:** 10.3389/fragi.2021.738512

**Published:** 2021-09-27

**Authors:** Alessandro Bitto, Nicole Tatom, Thomas Krivak, Peter Grotz, Matt Kaeberlein

**Affiliations:** Department of Laboratory Medicine and Pathology, University of Washington Medical Center, Seattle, WA, United States

**Keywords:** mTOR - mammalian target of rapamycin, C/EBP (CCAAT-enhancer-binding protein), diet-induced obesity, adefovir dipivoxil (ADV), metabolism and obesity

## Abstract

Aging and obesity are common risk factors for numerous chronic pathologies, and the compounding effects of old age and increased adiposity pose a serious threat to public health. Starting from the assumption that aging and obesity may have shared underpinnings, we investigated the antiobesogenic potential of a successful longevity intervention, the mTORC1 inhibitor rapamycin. We find that rapamycin prevents diet-induced obesity in mice and increases the activity of C/EBP-β LAP, a transcription factor that regulates the metabolic shift to lipid catabolism observed in response to calorie restriction. Independent activation of C/EBP-β LAP with the antiretroviral drug adefovir dipivoxil recapitulates the anti-obesogenic effects of rapamycin without reducing signaling through mTORC1 and increases markers of fat catabolism in the liver. Our findings support a model that C/EBP-β LAP acts downstream of mTORC1 signaling to regulate fat metabolism and identifies a novel drug that may be exploited to treat obesity and decrease the incidence of age-related disease.

## Introduction

The rate of obesity has been rising sharply in industrialized countries, especially in the United States (Hales et al., 2020), and poses a serious threat to already overburdened healthcare systems (Finkelstein et al., 2009; Cawley and Meyerhoefer, 2012). Obese individuals are at higher risk for the development of cardiovascular disease, hypertension, non-insulin-dependent diabetes, certain cancers, and metabolic syndrome (Keller and Lemberg, 2003; Wolin et al., 2010; Kachur et al., 2017). Importantly, the greatest risk factor for most of these diseases is old age (Kaeberlein, 2013), and obese older individuals are at even greater risk than either group alone (Samper-Ternent and Al Snih, 2012). It is estimated that 38% percent of the population of the United States over 60 years of age is obese, and another 40% of obese people aged 40–60 will enter geriatric age in the next 25 years (Flegal et al., 2016), defining a population at high risk for multiple comorbidities (Zhang et al., 2019).

The field of geroscience seeks to understand mechanisms that connect biological aging with age-related pathologies (Kennedy et al., 2014; Sierra, 2016). Several therapeutic approaches that target the molecular hallmarks of biological aging have been shown to increase lifespan and delay age-related pathologies in mice (Kaeberlein, 2017). Among these, inhibition of the mechanistic target of rapamycin (mTOR) has proven particularly robust and reproducible (Johnson et al., 2013; Kennedy and Lamming, 2016). Genetic knockdown of mTOR complex 1 (mTORC1) components or pharmacological inhibition of mTORC1 with rapamycin is sufficient to increase lifespan by up to 30% and delay or reverse functional declines of aging across many tissues and organs in mice including brain, heart (Wilkinson et al., 2012; Quarles et al., 2020), liver (Wilkinson et al., 2012), intestine (Yilmaz et al., 2012), immune system (Chen et al., 2009), tendon (Wilkinson et al., 2012; Zaseck et al., 2016), bone (Ma et al., 2018; An et al., 2020), skeletal muscle (Bitto et al., 2016; Joseph et al., 2019; Tang et al., 2019), auditory system (Altschuler et al., 2021), adipose (Mau et al., 2020), ovaries and endometrium (Wilkinson et al., 2012; Dou et al., 2017; Garcia et al., 2019), adrenal gland (Wilkinson et al., 2012), and the oral cavity (An et al., 2017; An et al., 2020).

C/EBP-β is a basic leucine zipper (bZIP) domain transcription factor highly expressed in liver, kidney, intestine, adipose, pancreatic islets, and innate immune cells. It regulates the expression of several genes involved in development, immune function, regeneration, differentiation, and metabolism (Guo et al., 2015; Pulido-Salgado et al., 2015). C/EBP-β is an intronless gene but it generates three isoforms with distinct yet not fully characterized functions, termed Liver Activating Protein* (LAP*), Liver Activating Protein (LAP), and Liver Inhibitory Protein (LIP) (Calkhoven et al., 2000). LIP, the shortest isoform, contains a bZIP DNA-binding domain but lacks a transactivation domain to recruit the RNA polymerase II complex. LAP and LAP* encode the full-length factor and contain a transactivation domain in addition to the bZIP and are considered functionally identical. Thus, LIP is considered a transcription inhibitor, while LAP and LAP* (hereafter referred to collectively as LAP) are regarded as transcription activators with essentially the same function.

The relative abundance of LAP and the ratio between LAP and LIP are regulated by mTORC1 through differential translation of the isoforms. The full length C/EBP-β mRNA contains a short inhibitory upstream open reading frame (uORF) prior to the LAP initiation site (Calkhoven et al., 2000). When mTORC1 activity is high, translation of the uORF is more likely, which causes the ribosome to skip over the LAP start site and favors translation of LIP. When mTORC1 activity is low, such as under nutrient depletion or in the presence of rapamycin, uORF translation is reduced, and translation of the full-length LAP is favored. Consistent with this model, pro-longevity interventions such as calorie restriction or rapamycin treatment increase LAP and reduce LIP expression in the liver of mice. Interestingly, genetic knockout of the uORF in mice (Cebpb^ΔuORF^) ablates expression of LIP, increases female lifespan (Müller et al., 2018), and improves several metabolic parameters including weight, adiposity, adipocyte size, energy expenditure, and adiponectin levels (Zidek et al., 2015).

The Nucleoside-analog Reverse Transcriptase Inhibitor (NRTI) adefovir dipivoxil has also been identified as a modulator of the LAP/LIP in a *in vitro* screening for compounds that reduce LIP translation (Zaini et al., 2017).

Since obesity and aging are overlapping risk factors for multiple pathologies, and rates of obesity increase with age (at least until about age 65), we set out to test whether the geroprotective intervention rapamycin could impact diet-induced obesity in adult mice. Here we report that mice treated with rapamycin are strongly protected against weight gain and fat accumulation associated with an obesogenic diet. Rapamycin-treated mice also show activation of C/EBP-β in the liver. Further, a small-molecule activator of C/EBP-β, adefovir dipivoxil, is sufficient to phenocopy the anti-obesogenic effects of rapamycin and to up-regulate enzymes involved in fatty acid metabolism in the liver. These observations support the model that mTORC1-inhibition prevents diet induced obesity by activation of C/EBP-β and identify adefovir dipivoxil as a potential therapeutic for obesity and aging.

## Materials and Methods

### Animals and Treatments

**FIGURE 1:**
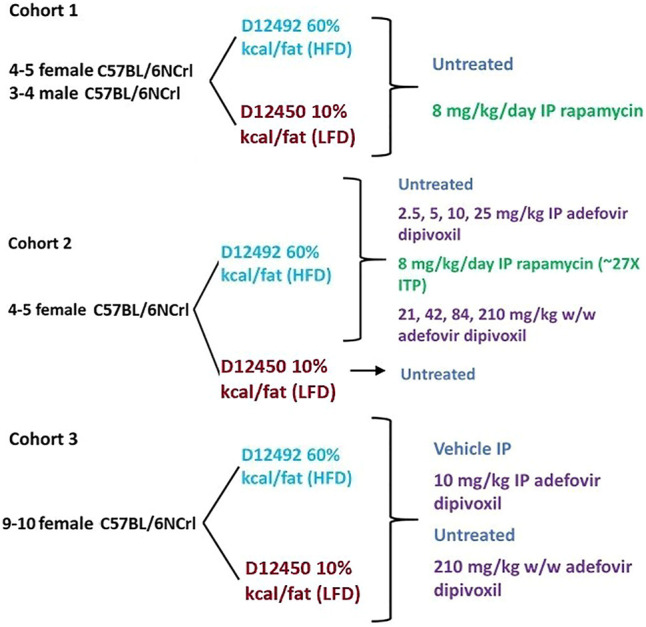
Experimental Layout**.** Experimental layout of the three cohorts of mice examined in this study. All animals were 8–9 months old at the time they were started on a diet/treatment regimen.

All experiments were conducted in agreement with the University of Washington Institutional Animal Care and Use Committee (protocol number PROTO201600017). 8–9 months old C57BL/6N Crl mice were obtained from Charles Rivers Laboratories. All animals were acclimated to the University of Washington’s animal facilities for 1 month before being enrolled in the study. Mice in cohort 1 (Figure 1) were housed in the Foege animal facility at the University of Washington. Male and female mice were housed in groups of three to five in Allentown JAG 75 cages. Animals were separated for fighting or excessive barbering. Mice in cohort two and three were housed in groups of three to five in Allentown NexGen Mouse 500 cages in the Animal Research Core Facility (ARCF) at the University of Washington. All animals were housed on a 14 h light, 10 h dark cycle. One week before the beginning of the study for each cohort, animals were randomly assigned to diets and treatments, although we ensured that initial weights were roughly equal among experimental groups.

Animals were fed either Research Diet D12492 (HFD) or D12450j (LFD) (Research Diets, New Brunswick NJ) and treated with daily intraperitoneal injections of 8 mg/kg rapamycin (LC Laboratories, Woburn MA) (cohort 1 and 2), daily intraperitoneal injections of 2.5 mg/kg, 5 mg/kg, 10 mg/kg, or 25 mg/kg adefovir dipivoxil (cohorts 2 and 3) (Carbosynth, UK), or with adefovir dipivoxil mixed in the chow for 6 weeks at 21 mg/kg, 42 mg/kg, 84 mg/kg, or 210 mg/kg w/w (cohorts 2 and 3). Rapamycin and adefovir dipivoxil stock solutions (100 mg/ml in DMSO) were diluted to their final concentrations in a water, 5% PEG-400, 5% Tween-80 vehicle.

Weight was monitored 3–5 times per week. Food consumption was monitored by giving around 150 g of food and weighing the remaining amount every 2–3 days to calculate weekly averages. At the end of the 6 weeks, all animals were fasted overnight and refed for 3 h at the beginning of the following day’s light cycle before cervical dislocation. Animals in cohort three received the last dose of adefovir dipivoxil upon being refed in the morning. Tissues were snap frozen in liquid nitrogen, fixed in neutral buffered formalin, or embedded in Tissue-Tek O.C.T. compound for further analyses. A diagram of the experimental setup is found in Figure 1.

### Body Composition

Body composition was measured at the beginning of the study and every 2 weeks until the end of the study using a EchoMRI 100H (EchoMRI, Houston TX).

### Western Blotting

Frozen tissues were ground in liquid nitrogen and protein were extracted in ice-cold RIPA buffer supplemented with protease and phosphatase inhibitors. 30°μg of protein extract were loaded onto NuPage Bis-Tris acrylamide gels (Thermofisher, Waltham MA), and transferred onto Immobilon P membranes (Millipore-Sigma, Burlington MA) with a BioRad Trans-Blot Turbo Transfer System (BioRad, Hercules CA). Membranes were blotted with antibodies against C/EBP-β (clone E299), Edil-3 (clone EPR12451), CPT-1a (clone 8F6AE9) (abcam, Waltham MA), ACSL-1 (clone D2H5), phospho-S6 (Ser 235–236, cat 2211), total S6 (clone 5G10), GAPDH (clone D16H11), beta-actin (clone 13E5) (Cell Signaling Technologies, Danvers MA).

### RT-PCR

RNA was extracted with the PureLink RNA mini kit (cat 12,183,018 Thermofisher, Waltham MA), and RT-PCR was performed with the iTaq universal SYBR Green One-Step Kit (Cat 172,510, BioRad, Hercules CA) on an Applied biosystem StepOne Plus Thermocycler (ThermoFisher, Waltham MA) according to the manufacturer’s specifications. Primer sequences for SCAD, MCAD, LCAD, VLCAD, and Actin were taken from (Zidek et al., 2015).

### Clinical Chemistry

Blood was collected upon cervical dislocation at the throat of the animals. Serum chemistry was performed by the Phoenix Central Laboratories (Mukilteo, WA). Due to the small sample size, testing was performed on a 1:8 dilution.

### Statistical Analysis

Unless otherwise noted, all data were analyzed by Student’s t test.

## Results

### Rapamycin Prevents Weight Gain and Fat Accumulation in Adult Mice and Activates Hepatic C/EBP-β

**FIGURE 2:**
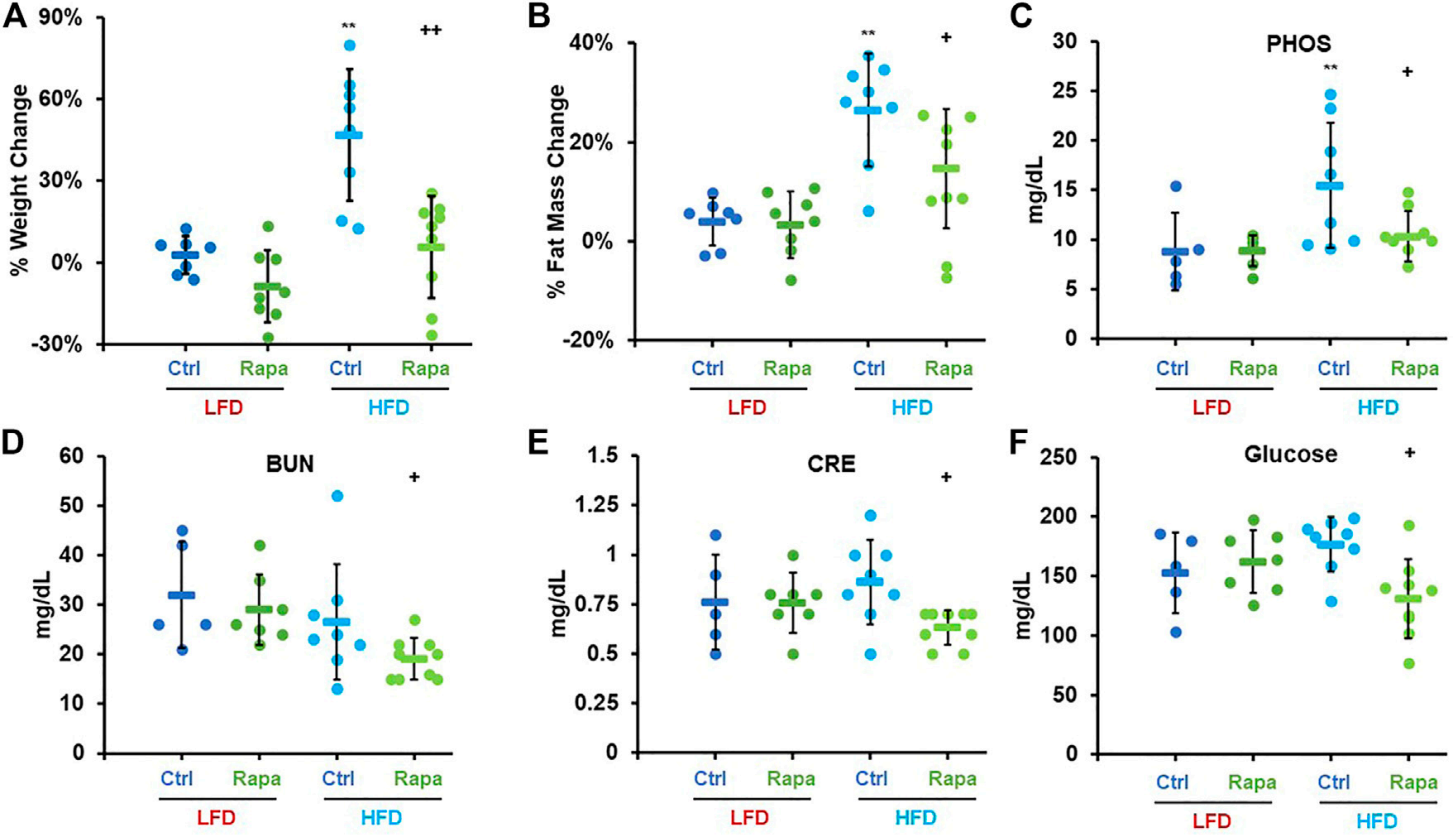
Rapamycin prevents diet-induced obesity in mice. Percent weight **(A)** and fat mass **(B)** change in mice fed either a low-fat diet (LFD) or a high-fat diet (HFD). Serum phosphate **(C)**, blood urea nitrogen **(D)**, creatinine **(E)**, and glucose **(F)** measured after a 24 h fast-3h refeed at the time of necropsy. PHOS: phosphate, BUN: blood urea nitrogen, CRE: creatinine, Ctrl: control, Rapa: rapamycin. Circles: individual animals, bars: average + - standard deviation. *: *p* < 0.05 vs LFD control, **: *p* < 0.01 vs LFD control, +: *p* < 0.05 vs HFD control, ++: *p* < 0.01 vs HFD control.

We monitored weight and fat mass over the course of 6 weeks in 8–9-month-old mice fed with either a diet containing 10% kcal/fat (LFD) or 60% kcal/fat (HFD) and treated with daily intraperitoneal (IP) injections of 8 mg/kg rapamycin. After 6 weeks, animals fed the HFD had gained almost 50% of their initial weight and accumulated an additional 40% of their original fat mass (Figures 2A,B). Conversely, animals that received daily injections of rapamycin gained barely any weight and accumulated significantly less fat, despite similar caloric intake (Supplementary Figure S1A). When analyzed by sex, female mice gained more weight and fat mass than males when fed the HFD regardless of treatment. Rapamycin was effective at significantly reducing both weight gain and fat accumulation in female mice alone (Supplementary Figures S1B,D), while in male mice we observed a trend toward decreased weight and fat accumulation (Supplementary Figures S1C,E *p* = 0.07 and 0.11 respectively) despite the reduced sample size. We performed a comprehensive metabolic panel at the end of the treatment period to determine whether mice on a HFD would show clinical signs of metabolic diseases (Table 1). To our surprise, most parameters were not significantly altered by diet, except for phosphate levels (Figure 2C). Rapamycin reduced hyperphosphatemia in HFD-fed mice and reduced the levels of other kidney dysfunction markers, such as blood urea nitrogen (BUN) and creatinine (CRE) (Figures 2D,E) Finally, rapamycin significantly reduced non-fasting blood glucose in HFD-fed animals (Figure 2F). Because of the larger magnitude of effect on weight and adiposity, we chose to perform additional studies in female mice.

**TABLE 1:** Serum metabolic panel at endpoint. GLU: Glucose. BUN: Blood Urea Nitrogen. CRE: Creatinine. CA: Calcium. PHOS: Phosphate. TP: Total Protein. ALB: Albumin. GLO: Globulin. A/G: Albumin/Globulin ratio. TBIL: Total Bilirubin. ALP: Alkaline Phosphatase. ALT: Alanine Transaminase. AST: Aspartate Transaminase. CHOL: Cholesterol. (*) *p* < 0.05 vs LFD control, (+) *p* < 0.05 vs HFD control.

**Test**	**Units**	**LFD control**	**LFD rapamycin**	**HFD control**	**HFD rapamycin**
GLU	mg/dL	153 ± 33.95	162.14 ± 26.39	176.75 ± 23.11	131.11 ± 33.36 (+)
BUN	mg/dL	32 ± 10.75	29 ± 7.12	26.5 ± 11.65	19.11 ± 4.2 (*)
CRE	mg/dL	0.76 ± 0.24	0.76 ± 0.15	0.86 ± 0.21	0.63 ± 0.09 (+)
CA	mg/dL	16.12 ± 22.08	7.26 ± 1.03	5.86 ± 2.05	6.77 ± 1
PHOS	mg/dL	8.83 ± 3.93	8.87 ± 1.55	15.46 ± 6.34 (*)	10.3 ± 2.52
TP	g/dL	6.18 ± 1.3	5.9 ± 0.59	6.98 ± 1.76	5.69 ± 0.33
ALB	g/dl	0.54 ± 0.36	1.16 ± 0.44 (*)	1.18 ± 0.62 (*)	1.29 ± 0.32 (*)
GLO	g/dL	5.64 ± 1.53	4.74 ± 0.9	5.8 ± 1.51	4.4 ± 0.35 (+)
A/G		0.12 ± 0.08	0.27 ± 0.11 (*)	0.21 ± 0.1	0.3 ± 0.09 (*)
TBIL	mg/dL	0.1 ± 0.12	0.13 ± 0.05	0.125 ± 0.07	0.11 ± 0.06
ALP	U/L	17.8 ± 34.8	79.71 ± 53.81 (*)	13.88 ± 16.51	15.22 ± 17.06
ALT	U/L	268.4 ± 243.94	159 ± 68.67	346.25 ± 223.23	249.56 ± 164.85
AST	U/L	1606.4 ± 1159.37	1390 ± 743.46	3239.25 ± 2369.13	1504.22 ± 771
CHOL	mg/dL	93 ± 58.94	130.57 ± 68.83	116 ± 42.40	202.33 ± 45.78 (* +)

**FIGURE 3:**
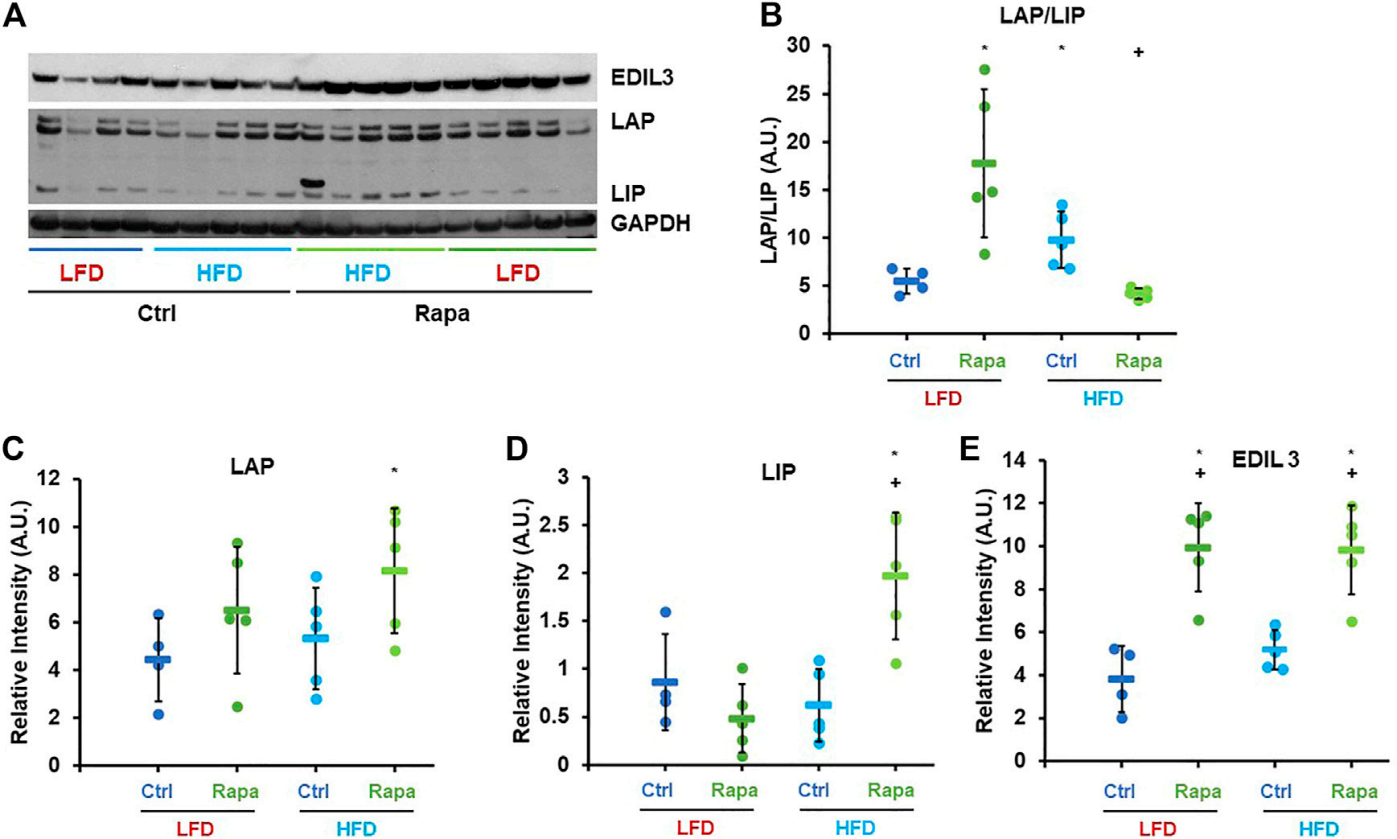
Rapamycin increases expression of C/EBP-β LAP and EDIL3. **(A)** Representative western blot image against EDIL3, C/EBP-β (LAP and LIP isoforms) and GAPDH. **(B–E)** Densitometric analysis of the western blot in panel **(A)** Circles: individual animals, bars: average + - standard deviation. *: *p* < 0.05 vs LFD control, **: *p* < 0.01 vs LFD control, +: *p* < 0.05 vs HFD control, ++: *p* < 0.01 vs HFD control.

Based on the previously described role of mTORC1 in C/EBP-β regulation, we investigated whether rapamycin increased expression of C/EBP-β targets in the livers of female mice fed a HFD via western blot. Consistent with previous reports (Zidek et al., 2015), rapamycin increased the ratio of the Liver Activating Protein (LAP) isoform to the shorter Liver Inhibitory Protein (LIP) in the liver of mice fed a LFD (Figures 3A,B). Surprisingly, in HFD-fed mice treated with rapamycin, the ratio of LAP/LIP was the lowest of all four experimental groups (Figures 3A,B). However, these animals showed the highest expression levels of both LAP and LIP isoforms and of the C/EBP-β-regulated gene EDIL-3 (Figures 3C–E) (Maekawa et al., 2015), indicating that rapamycin promotes the activity of C/EBP-β LAP in both LFD- and HFD-fed animals.

### Pharmacological Activation of C/EBP-β is Sufficient to Prevent Weight Gain Independently of mTOR Inhibition

**FIGURE 4:**
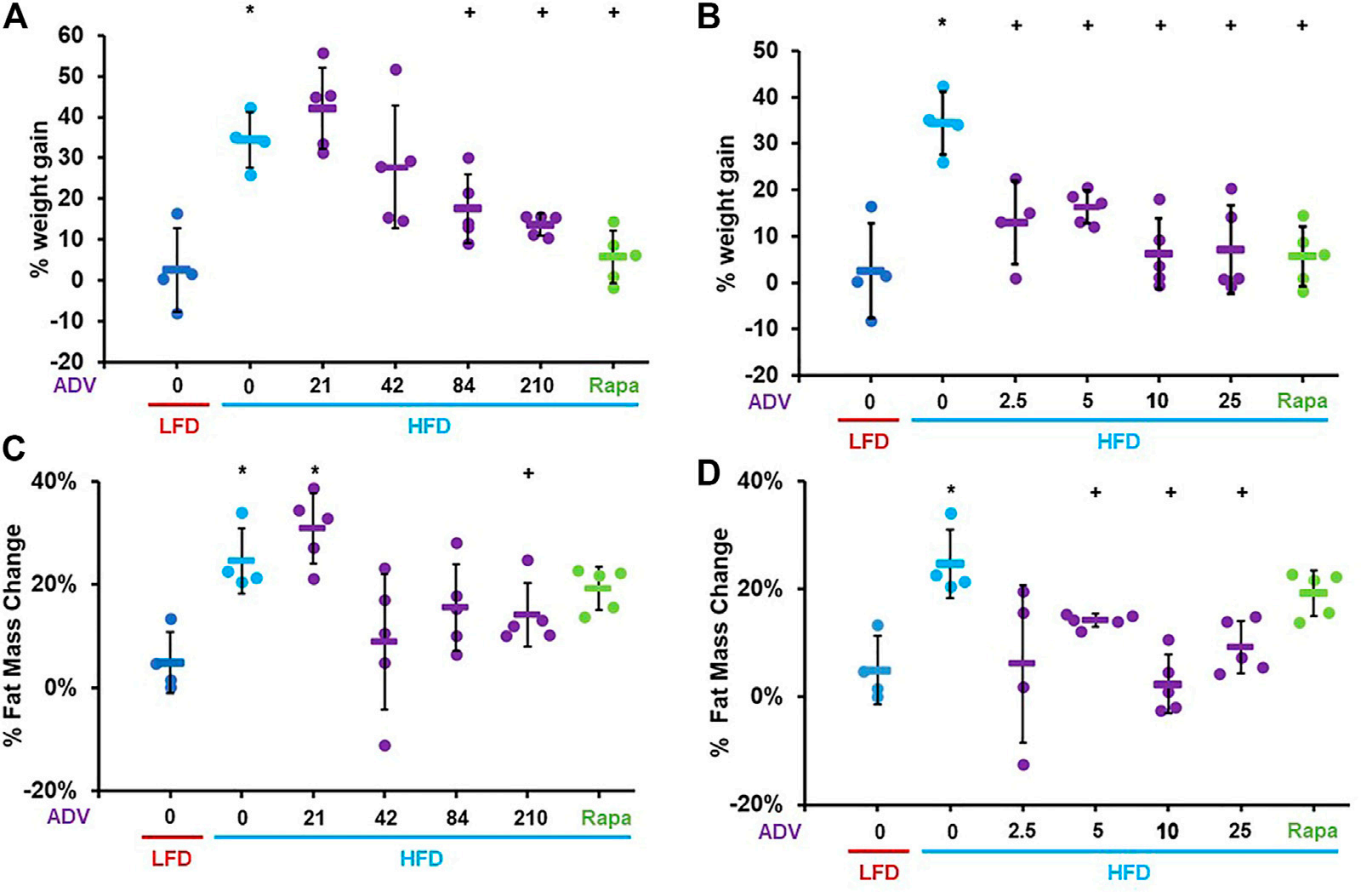
Adefovir dipivoxil prevents diet-induced obesity in mice. Percent weight change in mice fed either LFD or HFD and treated with increasing concentrations of adefovir dipivoxil (ADV) in the chow **(A)** or by intraperitoneal injection **(B)** Percent fat mass change in mice fed either LFD or HFD and treated with increasing concentrations of adefovir dipivoxil (ADV) in the chow **(C)** or by intraperitoneal injection **(D)** Circles: individual animals, bars: average + - standard deviation. *: *p* < 0.05 vs LFD control, **: *p* < 0.01 vs LFD control, +: *p* < 0.05 vs HFD control, ++: *p* < 0.01 vs HFD control.

To determine whether activation of C/EBP-β is sufficient to prevent diet-induced obesity in adult mice, we treated female mice with increasing doses of adefovir dipivoxil. Mice were treated with adefovir dipivoxil for 6°weeks in conjunction with a HFD challenge. We administered adefovir dipivoxil both orally, mixed in the chow, or via daily intraperitoneal injections and monitored weight and fat mass. Increasing doses of adefovir dipivoxil in the chow showed reduced weight accumulation at the end of the 6 weeks treatment (Figure 4A) with a dose of 210 mg/kg also showing significant reduction in the accumulation of fat mass (Figure 4C). Intraperitoneal injections of adefovir dipivoxil prevented weight gain at all concentrations tested and significantly reduced fat accumulation at 5, 10, and 25 mg/kg (Figures 4B,D). 10 mg/kg intraperitoneal adefovir dipivoxil treatment reduced diet-induced obesity to an even higher degree than rapamycin in this cohort.

We next asked whether adefovir dipivoxil inhibited mTOR signaling by assessing the phosphorylation status of ribosomal protein S6 at serine 235/236 and of AKT at serine 473 in the liver of these animals. Surprisingly, we found that HFD significantly reduced the levels of ribosomal protein S6 and AKT phosphorylation in the liver compared to LFD fed animals. As expected, rapamycin further decreased phosphorylation of ribosomal protein S6, but did not further decrease phosphorylation of AKT. In contrast, adefovir dipivoxil had no effect on ribosomal protein S6 phosphorylation nor any additional effects on AKT phosphorylation (Supplementary Figures S2B–D).

**FIGURE 5:**
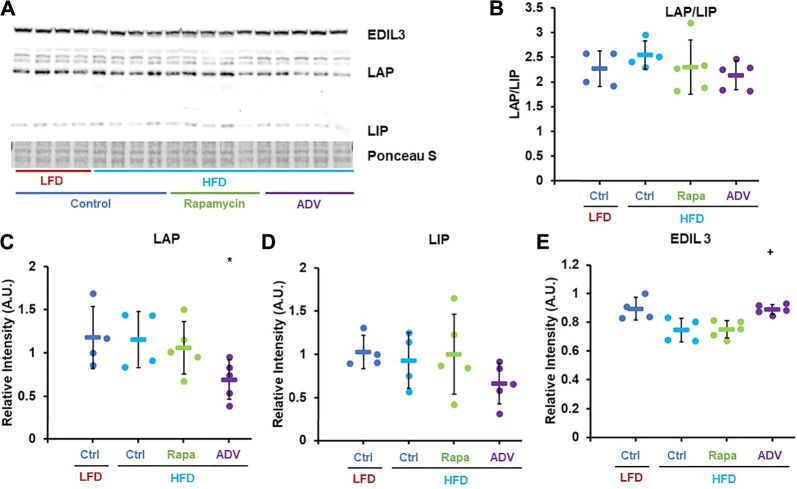
Adefovir dipivoxil increases expression of C/EBP-β LAP and EDIL3. **(A)** Representative western blot image against EDIL3, C/EBP-β (LAP and LIP isoforms) and GAPDH. **(B–E)** Densitometric analysis of the western blot in panel **(A)** Circles: individual animals, bars: average + - standard deviation. *: *p* < 0.05 vs LFD control, **: *p* < 0.01 vs LFD control, +: *p* < 0.05 vs HFD control, ++: *p* < 0.01 vs HFD control.

Next, we sought to assess whether adefovir dipivoxil increased expression or activity of C/EBP-β *in vivo*, as prior studies had only shown activation of C/EBP-β in cultured cells (Zaini et al., 2017). Contrary to our expectations, 6 weeks of treatment with adefovir dipivoxil reduced the levels of both LIP and LAP, without significantly altering the LAP/LIP ratio (Figures 5A–D) However, expression of the C/EBP-β target gene EDIL-3 was significantly elevated by adefovir dipivoxil (Figures 5A,E).

### Adefovir Dipivoxil Promotes the Expression of C/EBP-β-Target Genes Involved in Fatty Acid Oxidation

**FIGURE 6:**
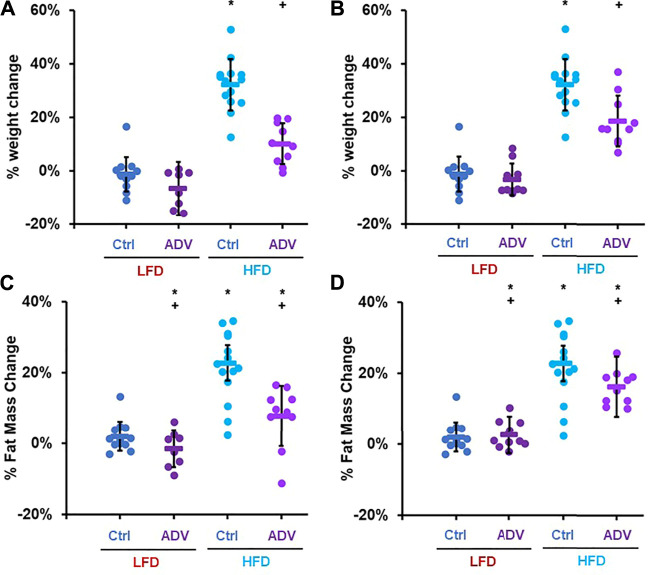
Anti-obesogenic effects of adefovir dipivoxil in mice. Percent weight change in mice fed either LFD or HFD and treated with 210 mg/kg adefovir dipivoxil (ADV) in the chow **(A)** or by intraperitoneal injection of 10 mg/kg/day ADV **(B)** Percent fat mass change in mice fed either LFD or HFD and treated with 210 mg/kg adefovir dipivoxil (ADV) in the chow **(C)** or by intraperitoneal injection of 10 mg/kg/day ADV **(D)** Circles: individual animals, bars: average + - standard deviation. *: *p* < 0.05 vs LFD control, **: *p* < 0.01 vs LFD control, +: *p* < 0.05 vs HFD control, ++: *p* < 0.01 vs HFD control.

To better characterize the anti-obesogenic effects of adefovir dipivoxil, we examined an additional cohort of female adult mice fed either LFD or HFD and treated with either 210 mg/kg adefovir dipivoxil in the diet or 10 mg/kg daily intraperitoneal injections of adefovir dipivoxil for 6 weeks. Consistent with our previous findings, both treatments reduced weight and fat accumulation (Figure 6). Importantly, we noticed no significant change in weight gain nor fat accumulation between animals receiving vehicle injections and untreated animals (Supplementary Figures S3A,B) despite slightly reduced caloric intake in HFD-fed animals receiving injections (Supplementary Figure S3C). Furthermore, animals receiving 10 mg/kg adefovir dipivoxil injection were resistant to diet-induced obesity despite higher caloric intake (Supplementary Figure S3C), indicating that the effects of adefovir dipivoxil are not due to food aversion or reduced appetite.

**FIGURE 7:**
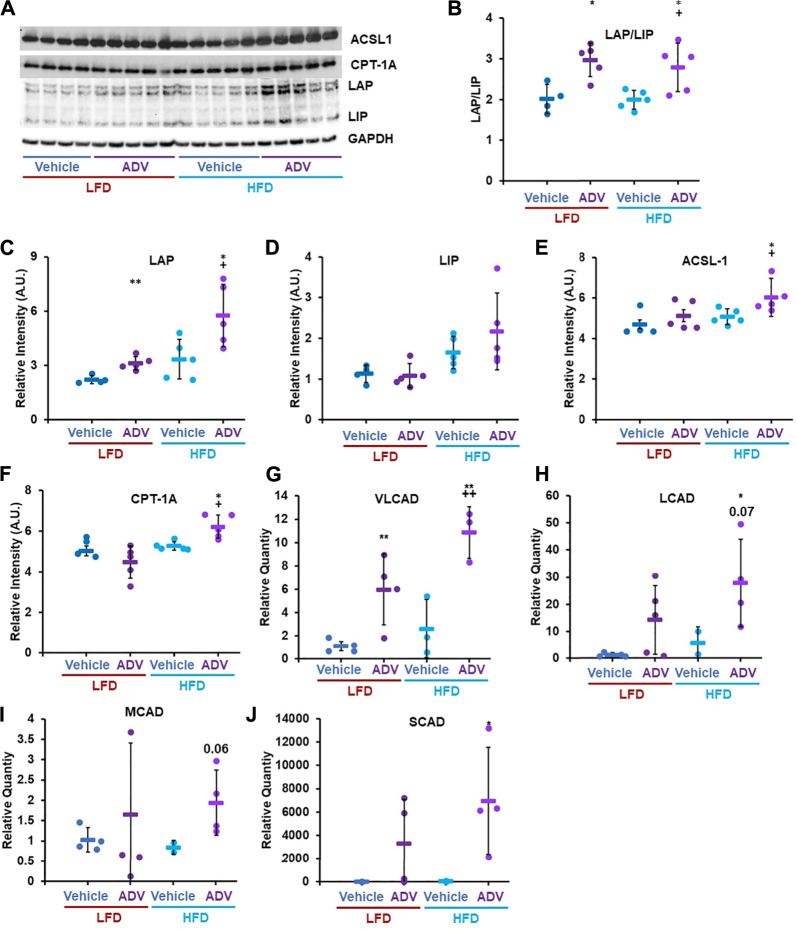
Adefovir dipivoxil promotes the expression of fatty acid oxidation genes. **(A)** Representative western blot image against C/EBP-β (LAP and LIP isoforms), ACSL1, CPT-1A, and GAPDH. **(B–F)** Densitometric analysis of the western blot in panel **(A)** Relative Quantity, as measured via RT-PCR, for VLCAD **(G)**, LCAD **(H)**, MCAD **(I)**, and SCAD **(J)** Circles: individual animals, bars: average + - standard deviation. *: *p* < 0.05 vs LFD control, **: *p* < 0.01 vs LFD control, +: *p* < 0.05 vs HFD control, ++: *p* < 0.01 vs HFD control.

The apparent disconnect between unaltered C/EBP-β LAP/LIP ratio with EDIL-3 activation in cohort two animals treated with adefovir dipivoxil (Figure 5) led us to hypothesize that the drug may have acute effects on C/EBP-β which are no longer apparent 24 h after treatment. To test this, we examined the acute effects of adefovir dipivoxil *in vivo*. At the end of the 6 weeks experimental period, animals in cohort three received a final injection of adefovir dipivoxil upon refeeding after an overnight fast and were sacrificed 3–5 h later. At this acute timepoint, adefovir dipivoxil increased the LAP/LIP ratio in the liver regardless of diet. This change was driven mostly by an increase in LAP in both LFD and HFD fed animals (Figures 7A–C). C/EBP-β LAP is known to increase fatty acid oxidation in the liver (Zidek et al., 2015; Zaini et al., 2017). We therefore quantified the expression levels of Acyl-CoA synthetase Long-chain family member 1 (ACSL1) and Carnitine-Palmitoyl Transferase 1A (CPT-1A), two proteins critical for the activation and transport of fatty acids into the mitochondria for oxidation. Adefovir dipivoxil increased the abundance of both enzymes in mice fed a HFD, consistent with increased fatty acid utilization (Figures 7A–F). We further examined whether adefovir dipivoxil shifted hepatic metabolism towards lipid catabolism by measuring the relative expression levels of all four fatty acid dehydrogenases via RT-PCR (Figures 7G–J). While a trend was visible for Long Chain (LCAD) and Medium Chain Fatty Acid Dehydrogenases (MCAD), adefovir dipivoxil significantly increased the levels of Very Long Chain and Short Chain Fatty (VLCAD) Acid Dehydrogenases (SCAD) in mice fed a HFD, further indicating increased fatty acid oxidation in these animals.

## Discussion

**FIGURE 8:**
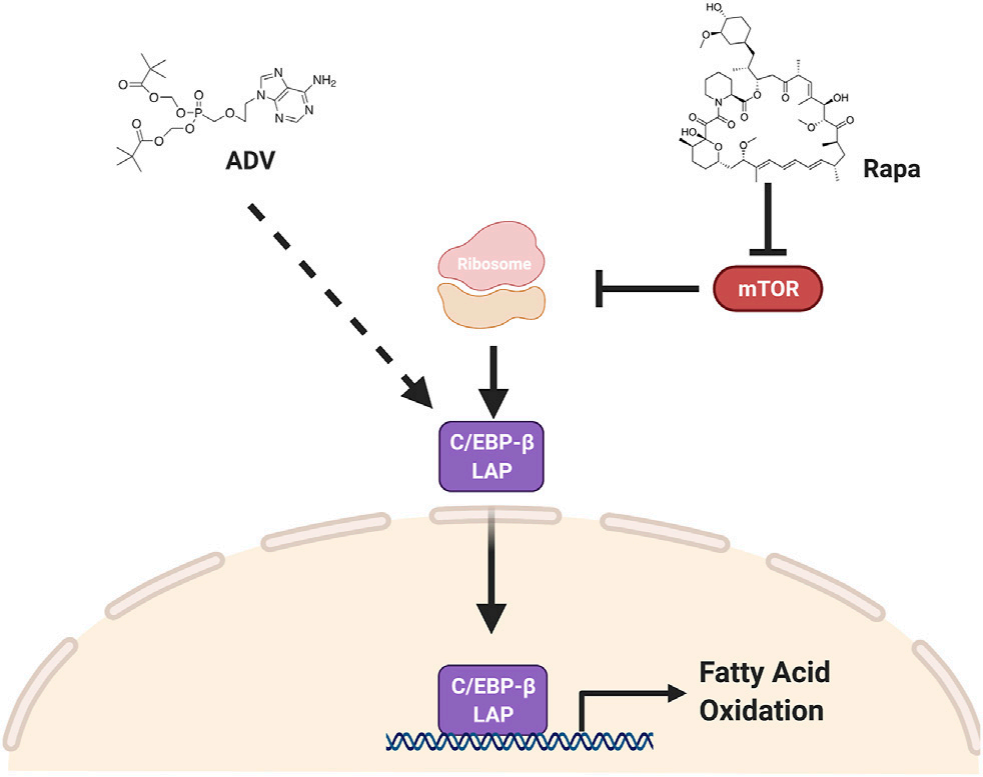
C/EBP-β LAP is a druggable target to increase fat catabolism. Two pharmacological interventions, rapamycin and adefovir dipivoxil, converge on C/EBP-β LAP to enhance fatty acid oxidation and increase fatty acid catabolism, thereby protecting against obesogenic diets. Rapamycin increases the relative expression of C/EBP-β LAP by increasing its translation, while adefovir dipivoxil is acting through a yet to be determined mechanism.

In this study, we provide evidence that inhibiting mTORC1 protects against diet-induced obesity by activating fatty acid catabolism through C/EBP-β. Furthermore, we show that adefovir dipivoxil is able to activate C/EBP-β *in vivo* and function as a potent anti-obesogenic agent. The effects of both rapamycin and adefovir dipivoxil cannot be attributed to reduced food consumption, but instead appear to be mediated, at least in part, through increased fatty acid oxidation. Taken together with prior studies, these observations suggest a model (Figure 8) whereby many of the metabolic effects of mTORC1 inhibition are likely mediated through activation of C/EBP-β, and that both mTOR and C/EBP-β are druggable targets to induce positive effects in the context of diet-induced obesity.

This work builds on prior studies which have explored targeting the mTOR pathway to prevent or reduce obesity in rodents, with mixed results. One study found that 14 ppm encapsulated rapamycin in the food exacerbated the effects of an obesogenic high-fat diet in 2 month-old male mice (Liu et al., 2014), while the same dose and route of administration showed beneficial effects in 2 month-old-male mice on a high-fat, high-sucrose diet (den Hartigh et al., 2018). This suggests that diet composition may impact the effects of mTORC1 inhibition on obesity. The diet used in this study was not high in sucrose and, interestingly, reduced mTORC1 signaling relative to the normal diet control (Supplementary Figure S2). Thus, it is not the case that carbohydrate composition alone accounts for these differences. Route of administration, bioavailability, and age likely all play significant roles in the anti-obesogenic effects of rapamycin.

Sex also appears to influence the efficacy of rapamycin as an anti-obesogenic treatment in mice. For example, 2 mg/kg/week intraperitoneal rapamycin was sufficient to decrease body weight in obese male mice (Chang et al., 2009), though oral gavage with 1 mg/ml rapamycin was only effective in female but not male 9 month-old mice on the same high fat diet used in our study. Though the small sample size likely prevented us from detecting a significant difference in weight and fat mass change in rapamycin-treated male mice on a HFD, we observed a clear trend (Supplementary Figure S1). There are numerous reports that female mice are more sensitive to rapamycin treatment and show higher blood levels of the drug at a given delivery concentrations (Harrison et al., 2009; Miller et al., 2011; Halloran et al., 2012; Wilkinson et al., 2012; Fok et al., 2014; Miller et al., 2014; Zhang et al., 2014; Fischer et al., 2015). Female mice also show consistently greater lifespan extension from rapamycin treatment at lower concentrations of the drug, but at higher concentrations male mice catch up to female mice and eventually show larger lifespan extension at the highest concentrations tested to date (Miller et al., 2014; Bitto et al., 2016). Thus, we speculate that rapamycin may have greater anti-obesogenic effects in male mice at other doses, and future studies should more carefully explore this question.

This study also expands on the relationship between mTOR signaling and regulation of metabolism by C/EBP-β. Prior work has shown that reduced mTORC1 signaling inhibits translation of C/EBP-β-LIP in mouse liver and adipose tissue, with subsequent activation of fatty acid catabolism and increased energy expenditure (Zidek et al., 2015). Our results are consistent with these findings, although under obesogenic conditions, rapamycin increased expression of both LAP and LIP isoforms as well as the expression of downstream effectors of C/EBP-β (Figures 3, 5). In addition to the potential differential regulation of C/EBP-β by mTORC1 under HFD conditions, it is also worth noting that the dose of rapamycin used in this study (8 mg/kg/day) is sufficient to inhibit both mTORC1 and mTORC2 (Martin-Perez et al., 2020). Inhibition of mTORC2 by chronic rapamycin treatment has been proposed to cause changes in glucose metabolism and insulin sensitivity in mice fed both normal and obesogenic diets (Liu et al., 2014), although the role of C/EBP-β has not been explored. It will be of interest to determine whether effects of rapamycin on C/EBP-β LAP are solely mediated by mTORC1, or if mTORC2 also plays a role, and how this influences the relationship between diet composition, adiposity, and fat metabolism. Notably, HFD alters AKT phosphorylation at serine 473 which could reflect changes in mTORC2 signaling in our study (Supplementary Figure S2). We did not detect any large changes in glucose homeostasis in response to rapamycin treatment (Figure 1), although we did not formally test glucose and insulin tolerance in these animals, nor can we completely rule out potential effects of either rapamycin or adefovir dipivoxil on alternative targets.

Like rapamycin, the effects of adefovir dipivoxil on LAP and LIP expression *in vivo* appear to be more complex than has been previously appreciated from *in vitro* studies. Adefovir dipivoxil was identified as a C/EBP-β activator from a cell-based screen for inhibitors of LIP translation that could mimic the effects of rapamycin (Zaini et al., 2017). Interestingly, we found that the drug decreased total abundance of both LIP and LAP without altering the ratio at a timepoint 24 h after the most recent treatment (Figure 5), but it significantly increased the expression of LAP (Figure 7) and the LAP/LIP ratio 3–5 h after treatment (Figure 7). Correspondingly, at both timepoints we observed increased expression of the C/EBP-β target gene EDIL3, and several additional C/EBP-β targets involved in fatty acid metabolism were induced at the 3–5 h timepoint . The mechanism by which adefovir dipivoxil is impacting C/EBP-β activity and fat metabolism remains unclear. Based on its nucleoside analogue structure, it is tempting to speculate that the drug could impair mitochondrial DNA replication and induce a mitohormetic response (Ristow and Zarse, 2010; Merry and Ristow, 2016); however, we currently have no direct evidence to support this hypothesis over alternative models.

Taken together, the various effects of rapamycin and adefovir dipivoxil observed here suggest that modulating the expression of both C/EBP-β isoforms, LAP and LIP, is likely critical for nutrient homeostasis. Mice lacking both isoforms are resistant to diet-induced obesity, but either die of severe hypoglycemia shortly after birth or live with severe metabolic imbalances (Croniger et al., 2001; Millward et al., 2007). Moreover, reintroduction of LIP in *Cebp* ^*−/−*^ animals restores adiposity and lipid homeostasis, including plasma leptin, free fatty acids, and triglycerides levels (Bégay et al., 2018), thus demonstrating that LIP has more than purely inhibitory function, as initially suggested by the lack of a transactivation domain (Calkhoven et al., 2000). C/EBP-β is highly expressed in liver, kidney, intestine, adipose, pancreatic islets, and innate immune cells and regulates the expression of several genes presiding over development, immune function, regeneration, differentiation, and metabolism (Guo et al., 2015; Pulido-Salgado et al., 2015). Tissue-specific transgenic models will be required to better understand the multiple effects of C/EBP-β isoforms on metabolism and nutrient homeostasis.

Beginning from a geroscience framework, we showed that reduced mTORC1 signaling prevents diet-induced obesity by increasing the activity of C/EBP-β. Furthermore, we identified adefovir dipivoxil as a potent activator of C/EBP-β *in vivo* and a potential anti-obesogenic therapeutic. Mice engineered to express only C/EBP-β LAP (*Cebp* ^*ΔuORF*^) show a marked reduction of age-related impairment and increased lifespan in female animals (Müller et al., 2018). It will be interesting to determine whether treatment with adefovir dipivoxil recapitulates the longevity phenotypes of *Cebp* ^*ΔuORF*^ animals. Nucleoside-analog reverse transcriptase inhibitors such as adefovir dipivoxil are well known for their mitochondrial toxicity and may be involved in HIV-associated neurodegeneration and other accelerated aging phenotypes (Payne et al., 2015); however, it is possible that at appropriate doses adefovir dipivoxil could be an effective drug for preventing obesity or delaying aging.

## Data Availability

The original contributions presented in the study are included in the article/Supplementary Material, further inquiries can be directed to the corresponding authors.
