# Supplementary material for: Evidence that C/EBP-β LAP Increases Fat Metabolism and Protects Against Diet-Induced Obesity in Response to mTOR Inhibition
Source: Front Aging. 2021 Sep 27;2:738512. doi: 10.3389/fragi.2021.738512 (PMC9261321; doi:10.3389/fragi.2021.738512)
Supplement: Supplementary file 1 [file DataSheet1.docx]

Supplementary Material

## Supplementary Figures

**
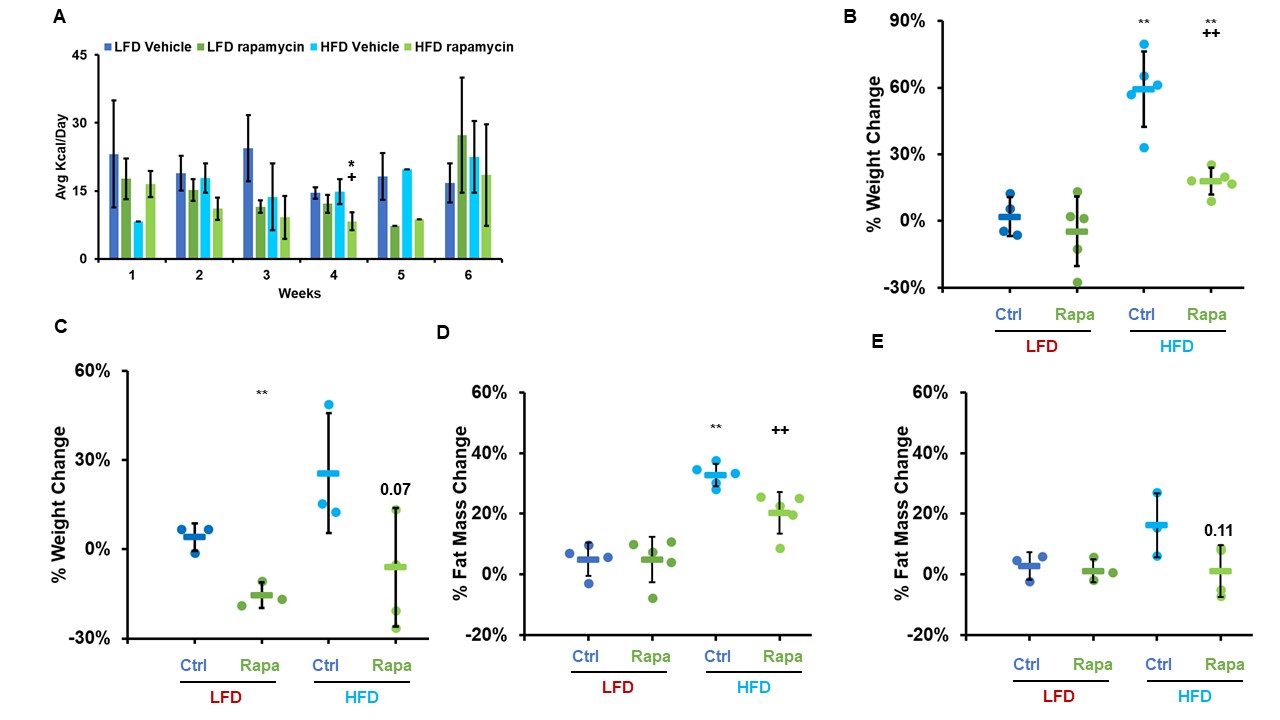
**

**Supplementary Figure 1.** **Rapamycin prevents diet-induced obesity in female mice.** A. Average caloric intake per week. Percent weight change in female (B.) and male (C.) mice fed either a low fat (LFD) or a high fat diet (HFD). Percent fat mass change in female (D.) and male (E.) mice fed either a LFD or a HFD.


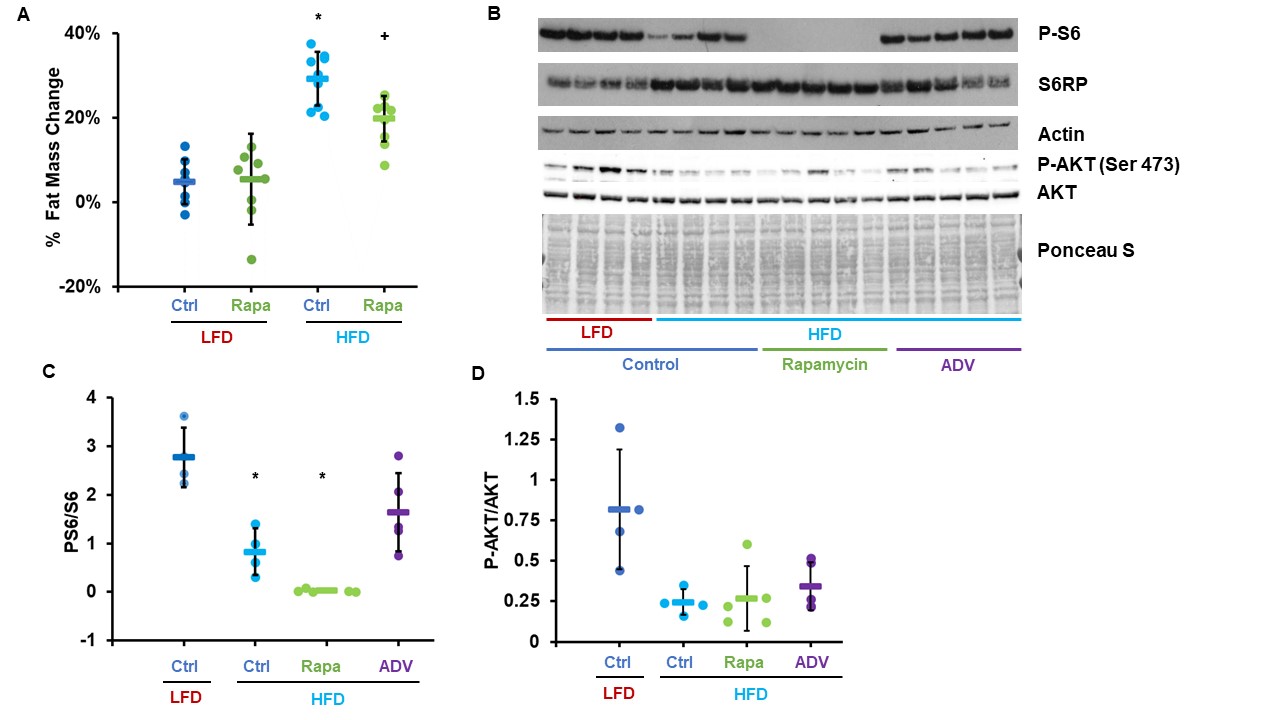


**Supplementary Figure 2. Adefovir dipivoxil does not inhibit mTOR signaling.** A. Percent fat mass change in female mice fed either a HFD or a LFD and treated with rapamycin, cohort 1 and 2 combined. B. Representative western blot and C. D. densitometric analysis of ribosomal protein S6 and AKT Ser473 phosphorylation in mice from cohort 2.


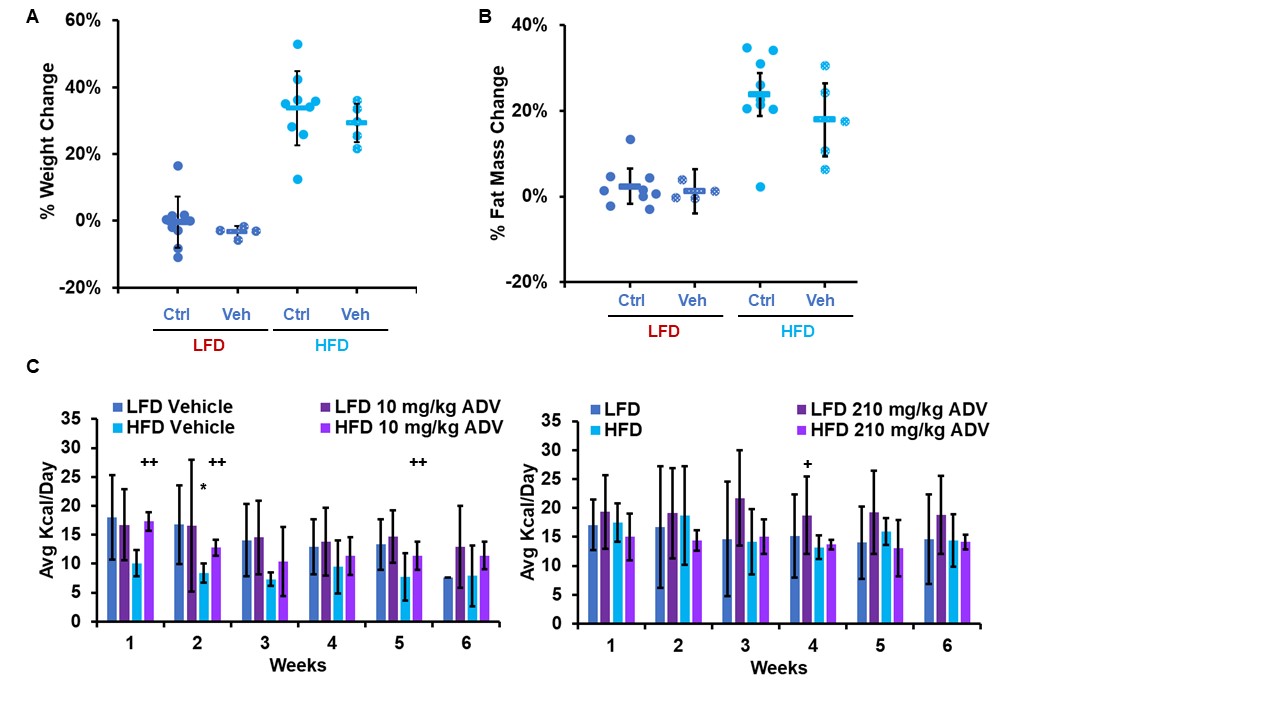


**Supplemental Figure 3. Adefovir dipivoxil injections increase caloric intake.** A. B. Percent weight and fat mass change in mice fed LFD or HFD and treated with vehicle injections or untreated. C. Caloric intake in mice fed LFD or HFD and treated with either 210 mg/kg adefovir dipivoxil in the chow or 10 mg/kg/day IP injections.
